# White-Matter Changes Correlate with Cognitive Functioning in Parkinson’s Disease

**DOI:** 10.3389/fneur.2013.00037

**Published:** 2013-04-12

**Authors:** Rebecca J. Theilmann, Jason D. Reed, David D. Song, Mingxiong X. Huang, Roland R. Lee, Irene Litvan, Deborah L. Harrington

**Affiliations:** ^1^Department of Radiology, University of California San DiegoLa Jolla, CA, USA; ^2^Research Service, VA San Diego Healthcare SystemSan Diego, CA, USA; ^3^Department of Neuroscience, University of California San DiegoLa Jolla, CA, USA; ^4^Neurology Service, VA San Diego Healthcare SystemSan Diego, CA, USA; ^5^Radiology Service, VA San Diego Healthcare SystemSan Diego, CA, USA

**Keywords:** Parkinson’s disease, cognition, diffusion tensor imaging, white-matter, cerebral cortex

## Abstract

Diffusion tensor imaging (DTI) findings from emerging studies of cortical white-matter integrity in Parkinson’s disease (PD) without dementia are inconclusive. When white-matter changes have been found, their relationship to cognitive functioning in PD has not been carefully investigated. To better characterize changes in tissue diffusivity and to understand their functional significance, the present study conducted DTI in 25 PD patients without dementia and 26 controls of similar ages. An automated tract-based DTI method was used. Fractional anisotropy (FA), mean diffusivity (MD), axial diffusivity (AD), and radial diffusivity (RD) were analyzed. Neuropsychological measures of executive functioning (working memory, verbal fluency, cognitive flexibility, inhibitory control) and visuospatial ability were then correlated with regions of interest that showed abnormal diffusivity in the PD group. We found widespread reductions in FA and increases in MD in the PD group relative to controls. These changes were predominantly related to an increase in RD. Increased AD in the PD group was limited to specific frontal tracks of the right hemisphere, possibly signifying more significant tissue changes. Motor symptom severity did not correlate with FA. However, different measures of executive functioning and visuospatial ability correlated with FA in different segments of tracts, which contain fiber pathways to cortical regions that are thought to support specific cognitive processes. The findings suggest that abnormal tissue diffusivity may be sensitive to subtle cognitive changes in PD, some of which may be prognostic of future cognitive decline.

## Introduction

Frontostriatal dysfunction is thought to be the basis for classic impairments in executive functions in Parkinson’s disease (PD) (Owen, 2004; Pagonabarraga and Kulisevsky, 2012). Most PD patients eventually develop dementia (Aarsland et al., 2003), which is up to five times more prevalent than in normal aging. With the development of neuroprotective agents that might delay disease progression, there is a need for markers sensitive to early neuronal pathology, when intervention might be most effective. Diffusion tensor imaging (DTI) is of keen interest as it is sensitive to microstructural tissue changes, especially in white-matter tracts, and is known to correlate with cognitive symptoms in other diseases.

The present study used DTI to investigate changes in cortical white-matter in non-demented individuals with PD relative to healthy controls. We also determined if abnormalities in fiber tracts correlated with cognitive functioning. DTI measures *in vivo* the local microstructural characteristics of water diffusion in tissues. The diffusion of water is extremely anisotropic, and is more restricted perpendicular than parallel to the fiber directions within axon bundles in the white-matter tracts. From DTI data, one can measure the diffusivity for the three principal axes of the diffusion tensor model (λ1 > λ2 > λ3), which can elucidate potential pathological processes associated with neurodegeneration. The most widely used measures of tissue integrity are mean diffusivity (MD) and fractional anisotropy (FA), which are the mean and normalized variance of the three principal axes, respectively (Basser et al., 1994). Low and high MD values respectively imply restricted and unrestricted diffusion. Higher FA values indicate that primarily diffusion occurs along one axis (i.e., coherent fiber bundle).

Although reduced FA is found in the basal ganglia and substantia nigra in PD without dementia (Yoshikawa et al., 2004; Menke et al., 2009; Modrego et al., 2011; Zhan et al., 2012), the findings from emerging studies of cortical white-matter integrity are inconclusive. Two whole-brain voxel-based DTI studies, each of 12 PD patients without dementia, reported FA decreases in frontal white-matter (Karagulle Kendi et al., 2008; Zhan et al., 2012). Another study reported changes in FA and in MD that were restricted to the cerebellum of PD patients without dementia (Zhang et al., 2011). In contrast, recent studies of 14–15 PD patients found widespread changes in FA and MD in anterior and posterior segments of cortical white-matter tracts (Rae et al., 2012; Gallagher et al., 2013). However, other studies of non-demented PD patients report normal diffusivity (Matsui et al., 2007b; Wang et al., 2011; Hattori et al., 2012; Kamagata et al., 2012). The reasons for conflicting findings are unclear, but may relate to the low signal/noise of most studies (e.g., one DTI dataset, limited diffusion-encoding directions), differences in DTI methods (Rae et al., 2012), and small sample sizes, which is problematic given the heterogeneity of symptoms in PD (Kehagia et al., 2010; Bohnen et al., 2012).

The behavioral significance of white-matter changes in PD is also not well understood. Increased motor symptoms are associated with reduced FA in the substantia nigra (Modrego et al., 2011; Prakash et al., 2012; Zhan et al., 2012) and the right splenium and forceps major (Rae et al., 2012), whereas a decline in olfactory functioning correlates with FA reductions in white-matter near the olfactory cortex (Ibarretxe-Bilbao et al., 2010). However, cognitive correlates of white-matter abnormalities have not been carefully studied in PD without mild cognitive impairment (MCI) or dementia. Performance on the Mini Mental State Examination (MMSE) does not correlate with FA in PD without dementia (Hattori et al., 2012; Kamagata et al., 2012). Though emerging studies suggest that white-matter abnormalities in PD correlate with executive functioning, the findings are conflicting. Reduced left parietal white-matter FA correlated with poorer performance in PD patients on a classic measure of executive functioning, namely the Wisconsin Card Sorting Task (WCST) (Matsui et al., 2007a). In contrast, reduced frontal white-matter FA correlated with worse performance on a composite measure of executive functioning and on the Stroop Interference test (Gallagher et al., 2013). While percentile rankings on composite measures of executive, attention, memory, and language functions also correlated with FA and/or MD in various segments of fiber tracts in PD patients (Zheng et al., 2013), it is unknown whether these results were related to abnormal tissue diffusivity in the tracts due to the absence of a control group.

The present study sought to build upon previous research by examining white-matter integrity and its cognitive correlates in a larger sample of PD patients (*n* = 25) than studied to date. We explored whether abnormal tissue diffusivity in PD was related to different facets of executive functioning (i.e., working memory, verbal fluency, cognitive flexibility, and inhibitory control) and visuospatial ability (Pagonabarraga and Kulisevsky, 2012). An automated voxel-based approach was used wherein FA values from the main white-matter tracts underlying the cortex of all subjects were realigned into standard space. This approach appears more sensitive to white-matter change in PD than whole-brain voxel-based analyses (Rae et al., 2012). Relative to other studies, our methods enhanced signal-to-noise by obtaining three DTI datasets and using 51 diffusion-encoding directions for subsequent averaging, which provides a more accurate spatial reconstruction of tissue structure due to the greater angular resolution (Behrens et al., 2007). As PD is associated with frontostriatal dysfunction, we predicted that pathology would be seen particularly in prefrontal cortex white-matter and would correlate in a regionally specific manner with different executive functions that emphasize processing in some different frontal networks. Posterior cortical changes were expected to correlate with visuospatial ability.

## Materials and Methods

### Participants and procedures

The protocol was approved by the University of California San Diego (UCSD) Human Research Protections Program. Written informed consent was obtained from all participants. Participants included 25 patients with idiopathic PD (14 males, 11 females) and 26 healthy adult controls (13 males, 13 females). PD patients exhibited at least two of the three cardinal features of the disorder, were levodopa responsive, and did not exhibit features of progressive supranuclear palsy, corticobasal degeneration, multiple systems atrophy, or dementia. Exclusionary criteria included metal in the head, neurological diagnoses other than PD, psychiatric diagnoses, history of alcohol or substance abuse, and major cognitive impairment as defined by a score of less than 26 on the MMSE (Table 1) (Folstein et al., 1975). All participants were right handed except for three PD patients. There were no group differences in age, years of education, and gender (Table 1). A movement disorder specialist assessed all PD patients when they were clinically on medication using the Unified Parkinson’s Disease Rating Scale (UPDRS) and the Hoehn and Yahr scale. Patients had a PD diagnosis for an average of 7.2 years. On the Hoehn and Yahr, 23 patients were stage 2 or 2.5 (mild bilateral disease) and two were stage 3.

**Table 1:** **Characteristics of Parkinson’s and control participants**.

	Parkinson’s		Controls		*t* Value	*p* Level
	Mean (SD)	Range	Mean (SD)	Range	
**Demographics**						
Age	68.0 (8.9)	52–84	65.9 (8.4)	51–82	<1.0	0.39
Education (years)	16.9 (2.2)	12–21	16.9 (2.7)	12–21	<1.0	0.99
**Mini Mental Status Examination**	28.7 (1.1)	27–30	29.3 (0.8)	27–30	2.2	0.04
**Working memory span**^1^
WAIS III: digits forward	11.4 (2.2)	6–14	10.9 (1.9)	7–14	<1.0	0.40
WAIS III: digits backward	7.4 (2.3)	4–13	7.8 (2.6)	4–14	<1.0	0.59
**Verbal fluency**^1^
DKEFS: letter	44.2 (11.3)	22–64	47.4 (12.4)	25–76	<1.0	0.39
**Cognitive flexibility**^2^
Trail Making Test Part A	37.5 (12.0)	22.6–77.9	32.0 (11.0)	16.0–70.3	−1.71	0.09
Trails Making Test Part B	90.7 (54.1)	36.0–279.3	75.5 (35.1)	41.1–183.3	−1.18	0.24
Part B minus Part A	53.2 (45.3)	4.0–201.4	43.4 (31.2)	9.0–143.2	<1.0	0.38
**Inhibitory control**^1^
Stroop Interference	34.0 (8.8)	20–49	38.6 (8.7)	23–53	1.73	0.09
**Visuospatial ability**^1^
Judgment of Line Orientation	11.6 (1.9)	8–14	12.4 (2.4)	6–15	1.32	0.19
**Disease characteristics**
Years diagnosis^3^	7.2 (4.8)	0.3–18.2				
UPDRS motor subscale^4^	25.4 (8.9)	11–40				
Hoehn and Yahr stage	2.36 (0.3)	2–3				
Levodopa dosage equivalence^5^	970.4 (986.8)	66.8–4850				

*Raw score means (standard deviations) and ranges are reported for all variables. Cognitive assessments were conducted when PD participants were taking their medication therapy. Twenty-one PD and 23 controls completed the Stroop Interference, the Letter Fluency from the Delis–Kaplan Executive Function System (DKEFS), and the Judgment of Line Orientation Tests. Data were available from the full sample on all other tests*.

*^1^Values are number correct*.

*^2^Values are time in seconds*.

*^3^Years diagnosis was unknown in one study participant*.

*^4^Total score on the motor subscale of the United Parkinson’s Disease Rating Scale (UPDRS)*.

*^5^Levodopa dosage equivalence was calculated using a method of Razmy et al. (2004)*.

Neuropsychological tests (Lezak, 1995) of executive functioning included measures of verbal working memory (Digits Span Forward and Backward from the WAIS III), verbal fluency [Letter Fluency test from Delis–Kaplan Executive Function System (DKEFS)], cognitive flexibility (Trail Making Test, Parts A and B), and inhibitory control (Stroop Interference). Visuospatial functioning was assessed by the Judgment of Line Orientation Test (JOLT).

### MRI procedures

Imaging was conducted at the UCSD Radiology Imaging Laboratory using a General Electric 1.5 T Signa HDx Twin Speed MRI system with an eight-channel head coil. T1-weighted high-resolution anatomic images were collected (Spoiled Gradient Recalled, TR = 10.6 ms, TE = 4.8 ms, FOV = 25.6, slice thickness = 1 mm, NEX = 1, Flip Angle = 10°). DTI images were acquired using a single-shot EPI sequence with diffusion-encoding along 51 directions, *b* value = 1000 s/mm^2^, one non-diffusion weighted image (*b_o_*), slice thickness = 2.5 mm, TR = 15.1 s, TE = minimum, matrix = 96 × 96 mm (automatically re-gridded onto a 128 × 128 matrix), FOV = 24 mms, and voxel size 1.875 × 1.875 × 2.5 mm. The enhanced number of diffusion-encoding directions relative to most other studies (6–12 gradient directions) allowed for more accurate spatial reconstruction of white-matter structure via the greater angular resolution. Subjects underwent three DTI acquisitions to increase the signal-to-noise ratio and to ensure at least one artifact-free scan. Due to discontinuation of testing, one control subject had one acquisition. All other subjects had three artifact-free scans.

### Diffusion tensor imaging analyses

Diffusion tensor imaging data were analyzed using FMRIB Software Library version 4.1.5 (FSL, http://www.fmrib.ox.ac.uk/fsl). For each subject, images from each DTI acquisition were concatenated and corrected for eddy currents by registering all brain volumes to the first non-diffusion weighted image using a rigid body transformation (no rotation). Each DTI scan was visually inspected to ensure the absence of susceptibility artifacts. Artifact-free DTI acquisitions were fit to a diffusion model for each voxel using FDT (Behrens et al., 2003). The diffusion tensor model was then diagonalized to determine the three eigenvalues of the tensor to calculate FA, MD, axial diffusivity (AD), and radial diffusivity (RD) maps. AD and RD measure tissue changes parallel (λ1) and perpendicular [(λ2 + λ3)/2] to the axonal tract, respectively.

Voxelwise statistical analyses of the DTI data were carried out using Tract-Based Spatial Statistics (TBSS) (Smith et al., 2006). First, a study-specific FA template image was generated by aligning all subjects’ FA data to a common space using a non-linear registration. TBSS automatically detects the most representative subject by identifying the FA image that requires the least amount of transformation to the FA images of all other subjects. From this FA template image, a skeleton was created that represented the center of all white-matter tracts common to all subjects. Given that FA steadily declines with age (Grieve et al., 2007), the skeleton was thresholded at FA >0.15 to include an analysis of white-matter tracts in an older population, which might be masked out using a higher threshold (e.g., >0.20). Each subject’s aligned FA data was then projected onto the skeleton and was analyzed by a voxelwise statistical analysis program (Nichols and Holmes, 2002). This process was repeated for MD, AD, and RD maps. Using the FSL Glm tool, a design matrix was constructed that included group, age, and gender. Age and gender were covariates of no interest. Age was mean-centered separately for each group. Five thousand Monte-Carlo permutations were conducted to establish voxel-by-voxel *p* values for tests of group differences in the DTI parameters. Threshold Free Cluster Enhancement (TFCE) (Smith and Nichols, 2009) was used to identify clusters for each parameter that significantly differed between the two groups (*p* < 0.05 familywise threshold).

### Regions of interest

To examine the functional significance of abnormal diffusivity in PD, we correlated the cognitive measures with the mean FA within regions of interest (ROI) generated from the voxels that showed significant group differences. We focused on FA because it is a stable metric (Bisdas et al., 2008; Teipel et al., 2011) that is more sensitive to white-matter diffusivity than other DTI measures (Papadakis et al., 1999), especially when a large number of diffusion directions are acquired (Rae et al., 2012). An advantage of the ROI approach is that it produces stable averages of parameters that may be more representative of the diffusion properties than a single voxel. ROI in each subject’s diffusion space were generated by deprojecting the thresholded voxels that showed significant group differences in FA in the voxel-based approach, and then binarized to create a mask. To account for anatomical differences among subjects, the white-matter parcellations from the FreeSurfer analysis stream (Fischl et al., 2002) were transformed into the individual diffusion space of each subject. Specifically, the Desikan atlas cortical parcellations, which are based on 34 conventional neuroanatomical regions defined by cortical sulci and gyri, were used to assign a label to the underlying white-matter via an automated FreeSurfer procedure (Salat et al., 2009). White-matter parcellations from conformed space were registered to the native FA space using an affine linear registration routine, FLIRT. The binary mask of the FA group difference was then multiplied by the white-matter parcellations to identify the corresponding region label for each subject, and the mean FA value was computed for each region. To generate ROI for abnormal white-matter that was not included in the Desikan atlas parcellations, the Johns Hopkins University (JHU) White-Matter Labels atlas was used (i.e., external capsule and anterior limb of internal capsule voxels). A small-volume correction was applied to the resulting ROI (i.e., minimum volumetric threshold of 140 mm^3^) to reduce the likelihood of false positives in the correlation analyses.

### Statistical analyses

To explore relationships between cognitive functioning and ROI that exhibited abnormal diffusivity in PD, cognitive measures were normalized to the control group by subtracting the individual raw scores for each measure from the control group mean and then dividing by the control group standard deviation. For the Trail Making Test, the raw score for Part A was subtracted from the raw score for Part B (Trails B-A), to obtain a measure of cognitive flexibility (i.e., set shifting) that adjusted for response speed. The resulting *Z* scores were then correlated with the FA in each ROI, adjusting for age and gender (partial correlations). As we had *a priori* hypotheses that measures of executive and visual spatial processing would respectively correlate with FA in tracts underlying the frontal or parietal-occipital cortices, a conventional *p* < 0.05 per comparison threshold was adopted. Nonetheless, the results should be considered exploratory and interpreted with caution due to the number of multiple correlations. SPSS version 18 was used for the partial correlation analyses.

## Results

### Neuropsychological test performance

Table 1 shows that MMSE scores were slightly lower in the PD than the control group. In both groups the lowest MMSE score was 27, indicating that no subject met the criteria for dementia. Significant group differences were not found on the neuropsychological tests.

### DTI voxelwise results

**Figure 1:**
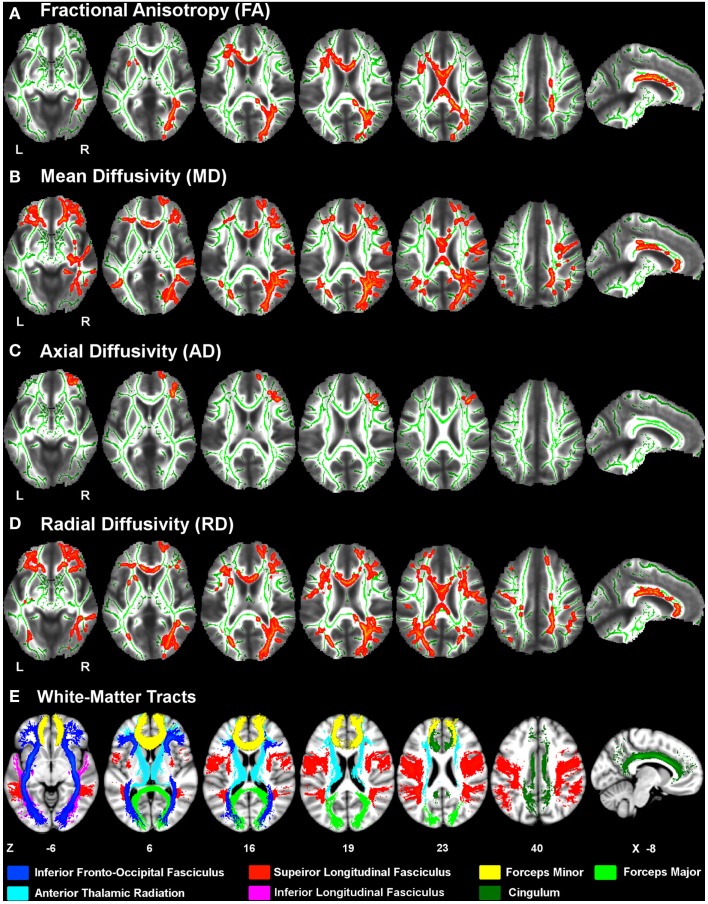
**White-matter diffusivity changes in Parkinson’s disease (PD)**. Axial and sagittal views of regions showing significantly *lower* fractional anisotropy **(A)** and *higher* mean diffusivity **(B)**, axial diffusivity **(C)**, and radial diffusivity **(D)** in the PD group relative to the control group (red clusters). The averaged FA-skeleton is displayed in green. Volumes of clusters showing significant change in PD were expanded on the figure to increase their visibility. White-matter tracts **(E)** showing significant diffusivity changes in PD were specified using the Johns Hopkins University (JHU) white-matter tractography atlas. Color labels for the JHU tracts are displayed at the bottom. *Z* coordinates (mm) of slices are from the Montreal Neurological Institute atlas. L, left hemisphere; R, right hemisphere.

Figure 1 displays the changes in tissue diffusivity in the PD group relative to the control group groups (*p* < 0.05 familywise threshold). The averaged FA-skeleton is shown in green. Volumes of abnormal tissue diffusivity are shown in red and were expanded to increase their visibility. The PD group showed significant reductions in FA and increases in MD and RD throughout anterior and posterior segments of the corpus callosum and white-matter tracts underlying the frontal, parietal, and occipital cortices. Table 2 shows that changes in FA, MD, and RD in PD were typically identified in both hemispheres and in the same tracts. Group differences in AD were found only in prefrontal cortex white-matter, where AD was increased in PD in the inferior fronto-occipital fasciculus, the forceps minor, and the anterior thalamic radiation.

**Table 2:** **White-matter tracts showing abnormal FA, MD, AD, and RD in Parkinson’s disease**.

Tract	FA	MD	AD	RD
Corpus callosum	B	B	–	B
Cingulum	B	B	–	B
Anterior corona radiata	L	B	–	B
Superior corona radiata	B	–	–	B
Anterior thalamic radiation	L^1^	B	R	B^1^
Forceps minor	B	B	R	B
Forceps major	R	B	–	B
Inferior fronto-occipital fasciculus	B^2^	B^3^	R	B^2^
Superior longitudinal fasciculus	B	B	–	B
Inferior longitudinal fasciculus	R	R	–	B

Total voxels	6068	19431	818	19125


*Fractional anisotropy (FA) was reduced and mean diffusivity (MD), axial diffusivity (AD), and radial diffusivity (RD) were increased in the Parkinson’s group. B, bilateral hemispheres; L, left hemisphere; R, right hemisphere*.

*^1^The tract includes the left internal capsule*.

*^2^The tract includes the left external capsule*.

*^3^The tract includes the right external capsule*.

### Correlation of cognitive measures with abnormal diffusivity

Table 3 lists the 28 ROI that were derived from the voxel-based analyses that uncovered group differences in FA. ROI were comprised of the corpus callosum, frontal, parietal, and occipital white-matter in both hemispheres, and subcortical fiber tracts (left external capsule and anterior limb of the internal capsule). An exception was the right hemisphere dominance for parietal and occipital ROI, owing to the criterion adopted for selecting only larger ROI (i.e., ≥140 mm^3^) for the correlation analyses.

**Table 3:** **Regions of interest (ROI) derived from voxelwise tests of group differences**.

Region name	FA volume (mm^3^)
**Corpus callosum**	
[1] Mid-anterior	235.19
[2] Central	256.99
[3] Mid-posterior	240.47
**Frontal**	
[4] L caudal anterior cingulate	1428.75
[5] R caudal anterior cingulate	1253.67
[6] L anterior corona radiata^1^	374.76
[7] L superior corona radiata^1^	142.73
[8] L superior frontal	480.58
[9] R superior frontal	332.23
[10] L rostral middle frontal	1142.93
[11] L caudal middle frontal	192.30
[12] L pars triangularis	320.80
[13] L pars opercularis	323.44
**Parietal**	
[14] L paracentral	187.50
[15] R paracentral	621.91
[16] L posterior cingulate	2215.19
[17] R posterior cingulate	1874.53
[18] L isthmus cingulate	359.30
[19] R isthmus cingulate	740.04
[20] R precuneus	2548.12
[21] R superior parietal	1872.07
[22] R inferior parietal	2543.55
**Occipital**	
[23] R cuneus	364.57
[24] R pericalcarine	548.79
[25] R lingual	203.29
[26] R lateral occipital	1912.50
**Subcortical**	
[27] L external capsule^1^	519.96
[28] L anterior limb of internal capsule^1^	205.31

*White-matter in the above ROI showed significant group differences in FA. Region labels typically correspond to white-matter underlying cortical areas from the Desikan atlas*.

*^1^The Johns Hopkins University (JHU) white-matter labels and tractography Atlas was used to generate ROI for voxels showing significant group differences in white-matter that was not included in the Desikan atlas white-matter parcellations*.

**Figure 2:**
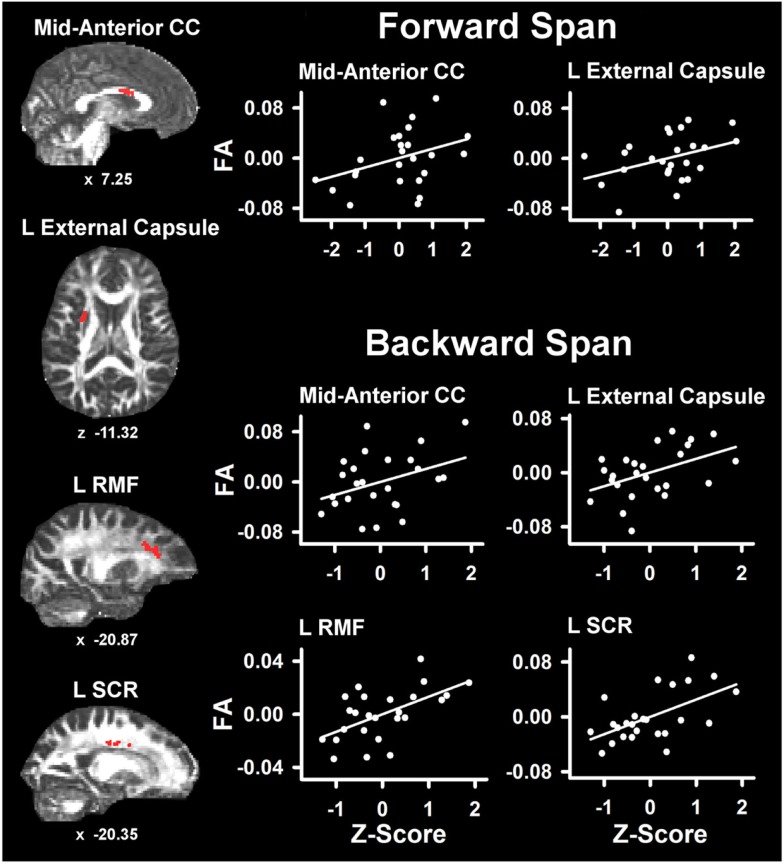
**Partial correlations between verbal working memory and fractional anisotropy (FA) in PD**. Scatter plots show the relationship between Digit Span Forward and Digit Span Backward to FA in representative ROI. The values plotted for all measures are residuals from the regression of age and gender onto regional FA and cognitive performance. Cognitive measures were normalized to the control group by subtracting the individual raw scores for each measure from the control group mean and then dividing by the control group standard deviation. Higher *Z* scores reflect better performance. The spatial location of FA in each ROI is displayed in diffusion space for representative individual subjects. L, left hemisphere; CC, corpus callosum; RMF, rostral middle frontal; SCR, superior corona radiata. Coordinates designate distance in mm from anterior commissure: *x*, left (−)/right (+) and *z*, superior (+)/inferior (−).

**Figure 3:**
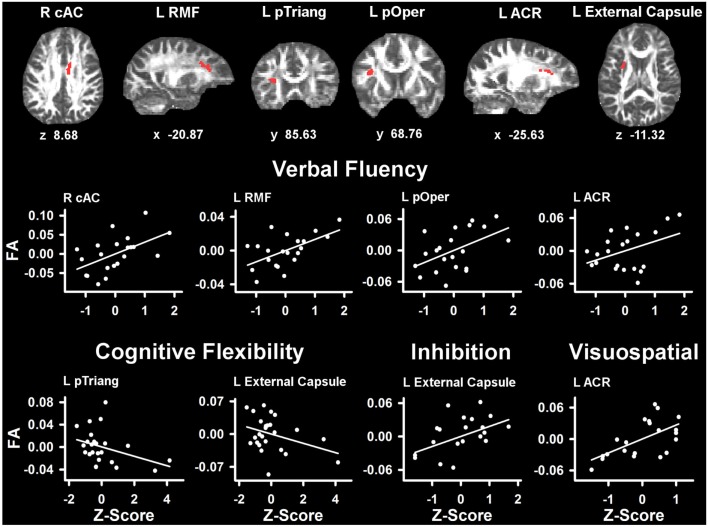
**Partial correlations between tests of executive and visuospatial functioning and fractional anisotropy (FA) in PD**. Scatter plots show the relationship between verbal fluency, cognitive flexibility (Trails B-A), inhibition (Stroop Interference), and visuospatial (JOLT) functioning and FA in representative ROI. The values plotted for all measures are residuals from the regression of age and gender onto regional FA and cognitive performance. Cognitive measures were normalized to the control group by subtracting the individual raw scores for each measure from the control group mean and then dividing by the control group standard deviation. Higher *Z* scores reflect better performance, except for cognitive flexibility where higher values signify worse performance (i.e., longer time to complete Trails Part B after subtracting Part A performance). The spatial location of FA in each ROI is displayed in diffusion space for representative individual subjects. L, left hemisphere; R, right hemisphere; ARC, anterior corona radiata; cAC, caudal anterior cingulate; pOper, pars opercularis; pTriang, pars triangularis; RMF, rostral middle frontal. Coordinates designate distance in millimeters. from anterior commissure: *x*, left (−)/right (+); *y*, anterior (+)/posterior (−); and *z*, superior (+)/inferior (−).

Table 4 lists the ROI that showed significant partial correlations (i.e., adjusted for age and gender) with the cognitive measures. Scatter plots of selected correlations are shown in Figures 2 and 3, together with representative examples of the ROI in diffusion space for individual PD patients. Plots graph the residuals of the cognitive and FA measures, after adjusting for age and gender. Figure 2 shows that poorer forward and backward working memory span was associated with lower FA in the mid-anterior corpus callosum and the left external capsule. Poorer backward span also correlated with lower FA in left hemisphere frontoparietal white-matter (rostral middle frontal cortex, superior corona radiata, and paracentral lobule). Verbal fluency performance correlated with lower FA especially in frontal regions (mid-anterior corpus callosum, bilateral caudal anterior cingulate, left anterior corona radiata, rostral middle frontal cortex, pars opercularis) (Figure 3), but also parietal white-matter (left paracentral lobule). Poorer cognitive flexibility (higher score signifies worse performance) correlated with lower FA in the right pericalcarine cortex and the left pars triangularis and external capsule (Figure 3). Poorer inhibitory control and visuospatial ability respectively correlated with lower FA in the left external capsule and left anterior corona radiata (Figure 3).

**Table 4:** **Partial correlations of FA with cognitive measures in Parkinson’s disease**.

	Verbal working memory		Verbal fluency	Cognitive flexibility	Inhibition	Visuospatial
	Forward	Back	Letter	Trails B-A	Stroop	JLOT
**FA**						
**Corpus callosum**						
[1] Mid-anterior	0.355*	0.367*	0.401*			
**Frontal**						
[4] L caudal anterior cingulate			0.457**			
[5] R caudal anterior cingulate			0.539***			
[6] L anterior corona radiata			0.404*			0.605***
[7] L superior corona radiata		0.565***				
[10] L rostral middle frontal		0.568***	0.570***			
[12] L pars triangularis				−0.368*		
[13] L pars opercularis			497**			
**Parietal**						
[14] L paracentral		0.454**	0.442*			
**Occipital**						
[24] R pericalcarine				−0.466**		
**Subcortical**						
[27] L external capsule	0.394*	0.453**		−0.356*	0.422*	

*p < 0.05, **p < 0.025, ***p < 0.01.

**Z* scores for each measure were correlated with FA in each ROI, adjusting for age and gender. Numbers in brackets correspond to the ROI listed in Table 3. Back, backward span; JLOT, Judgment of Line Orientation*.

To determine if the cognitive-FA relationships were specific to PD, *post hoc* partial correlation analyses were conducted in the control group for same ROI that significantly correlated with cognitive measures in the PD group. All correlations were non-significant except for the left paracentral lobule, which correlated with verbal fluency (*r*_partial_ = 0.42, *p* < 0.05).

### Correlation of clinical measures of disease severity with abnormal diffusivity

We also conducted partial correlations of FA with clinical measures of disease, adjusting for age and gender. Disease duration and levodopa dosage equivalence did not correlate with FA (*p* > 0.05). The UPDRS total motor score correlated negatively with FA in white-matter underlying the right pericalcarine cortex (*r*_partial_ = −0.516, *p* < 0.01), even when disease duration (*r*_partial_ = −0.471, *p* < 0.02) and levodopa dosage equivalence (*r*_partial_ = −0.483, *p* < 0.02) were added as covariates.

## Discussion

The present study uncovered several important findings. First, FA was decreased throughout the corpus callosum and in the frontal, parietal, and occipital white-matter of both hemispheres. A novel finding was that FA changes were largely due to increased in diffusivity perpendicular to the axon (RD), whereas increased diffusivity parallel to the axon (AD) was restricted to specific frontal tracts of the right hemisphere. Importantly, despite the absence of significant cognitive deficits in the PD group, abnormal FA in PD correlated in a regionally specific ways with performances in different cognitive domains, but not with clinical indices of motor symptom severity. Moreover, these FA-cognitive relationships were not found in the control group, suggesting that abnormal white-matter diffusivity may be sensitive to pathological processes that influence cognition in PD.

### Abnormal diffusion patterns in PD

The FA reductions throughout anterior and posterior segments of fiber tracts and the corpus callosum is supported by recent reports (Rae et al., 2012; Gallagher et al., 2013), but contrasts with other findings of normal FA (Matsui et al., 2007b; Boelmans et al., 2010; Wang et al., 2011; Hattori et al., 2012; Kamagata et al., 2012) or regionally circumscribed FA reductions (Karagulle Kendi et al., 2008; Zhang et al., 2011; Zhan et al., 2012) in PD without dementia. These discrepancies may partly relate to our larger sample size relative to most other studies, which is important given the considerable variability in clinical phenotypes. Our findings may also relate to the enhanced signal-to-noise of our imaging protocol and the skeleton-based analysis, which can provide a more accurate subject-by-subject alignment of tracts (Rae et al., 2012), especially in older adults where some degree of atrophy is common.

Fractional anisotropy reductions in PD were largely due to elevated RD. Changes in RD with unchanged AD have been attributed to myelin loss (Song et al., 2002; Schmierer et al., 2007; Klawiter et al., 2011), yet demyelination is not a significant pathogenic process in PD (Teismann and Schulz, 2004). Rather, alpha-synuclein pathology is characteristic of PD. However, there are also considerable early changes in glia (Halliday and Stevens, 2011). Microglia play a role in PD neuropathogenesis, including activation from alpha-synuclein aggregation (Teismann and Schulz, 2004). Activation of microglia accompanies demyelination (Song et al., 2005), suggesting that microglia may be involved in causing the changes in RD. Though histological validation is needed, RD increases in PD may reflect early stages of degeneration that are related to alpha-synuclein accumulation and the activation of microglia. This may alter the compactness of the axonal fibers, thereby increasing RD (Smith et al., 1981).

The preponderance of RD changes in PD without dementia contrasts with Alzheimer’s disease and to a lesser extent MCI, wherein decreased FA throughout the brain often relates to changes in both RD and AD (Bosch et al., 2012). This is relevant to our finding of increased AD in some right hemisphere frontal tracts, in which RD was also increased. Pathological processes that cause changes in AD and RD often occur in close proximity (Song et al., 2003), and may signify more significant early cell loss and gliosis than RD changes by themselves. This possibility is consistent with our hypothesis that pathological changes would be present particularly in prefrontal cortex white-matter.

### Functional significance of white-matter changes

Individual differences in neuropsychological test performance in the PD group were associated with tissue diffusivity especially in anterior segments of the left hemisphere frontal white-matter and the external capsule, which contain cortico-cortical association fibers. These findings were not related to motor symptom severity, disease duration, and LDE, which did not correlate with tissue diffusivity. This agrees with a study of 12 non-demented PD patients, wherein a worsening of motor symptoms correlated only with FA reductions in the putamen and substantia nigra (Zhan et al., 2012), which were not evaluated in our study.

We found that verbal fluency was associated with FA in the mid-anterior corpus callosum, a main source for interhemispheric communication. Verbal fluency also correlated with FA in multiple frontal white-matter regions including the left rostral middle frontal cortex (i.e., dorsolateral prefrontal cortex) and Broca’s area (left pars opercularis). These results concur with reports of largely frontal activation during verbal fluency in young adults (Abrahams et al., 2003; Birn et al., 2010), verbal fluency deficits after frontal lobe damage, especially to the left hemisphere (Baldo et al., 2006; Robinson et al., 2012), and improved verbal fluency in PD after repetitive transcranial magnetic stimulation to the left dorsolateral prefrontal cortex (Pereira et al., 2013).

Verbal working memory also correlated with FA in the mid-anterior corpus callosum. In addition, backward span correlated with FA changes near the left rostral middle frontal cortex and the superior corona radiata and paracentral lobule, which contain fibers near the supplementary motor area, the cingulate gyrus, and the parietal cortex. Frontostriatal and parietal networks govern working memory, especially when executive functions are engaged to manipulate the contents of active memory (Lewis et al., 2004; Badre, 2008; McNab and Klingberg, 2008), as for the backward span test. The current findings are compatible with a functional imaging study that reported reduced frontostriatal activation in PD individuals with executive impairment relative to a cognitively unimpaired PD group when manipulating information in working memory (Lewis et al., 2003).

Interestingly, FA in the external capsule correlated with most executive functioning measures including forward and backward working memory span, inhibitory control (Stroop Interference), and cognitive flexibility (Trails B-A). It has been suggested that the external capsule is important for these functions, because it carries cholinergic fibers from the basal forebrain to the cerebral cortex. Indeed, a recent study reported that cholinergic denervation in PD without dementia correlated with working memory, inhibitory control, and executive functioning, but not with visuospatial processing (Bohnen et al., 2012). These findings were independent of the relationships found between dopamine denervation and the same cognitive measures (Bohnen et al., 2012), consistent with the involvement of multiple neurotransmitter systems in PD (Kehagia et al., 2010).

Trails B-A performance was uniquely associated with FA in the pathway beneath the right pericalcarine cortex and the left pars triangularis, which is near Broca’s area. These results are functionally meaningful because set shifting on this task emphasizes visual search and engages subvocalization process. Lastly, we found that visuospatial ability (Judgment of Line Orientation) correlated strongly with FA in the left anterior coronal radiata, possibly because optimal performance partly depends on prefrontal control processes that govern attention and the integration of relational information (Watson and Chatterjee, 2012). Contrary to our hypothesis, visuospatial processing was not related to the diffusivity of tissue near posterior segments of the cortex, despite significant FA abnormalities in these tracts. The reason for this is unclear, but other tests of visuospatial abilities may better probe for posterior cortical functioning.

## Conclusion

The present results suggest that microstructural changes in white-matter may be sensitive to subtle cognitive decline, some of which may be prognostic of future cognitive deterioration. This contrasts with white-matter hyperintensities in PD, which do not correlate with cognitive functioning even in PD patients with MCI (Dalaker et al., 2009). Future research should explore relationships between white-matter diffusivity and functioning in other cognitive domains including on tests of posterior cortical functioning, which may be more prognostic of risk for dementia than performance on tests of frontal-based executive functions (Williams-Gray et al., 2009; Pagonabarraga and Kulisevsky, 2012). The functional significance of diffusivity changes in PD may also be better characterized via tractography techniques due to their neuroanatomical specificity, which may translate into more robust relationships with functioning in different cognitive domains.

## Conflict of Interest Statement

The authors declare that the research was conducted in the absence of any commercial or financial relationships that could be construed as a potential conflict of interest.
